# Association between a reduction in triglyceride levels and risk of cardiovascular events

**DOI:** 10.1016/j.ahjo.2025.100647

**Published:** 2025-10-16

**Authors:** Izuki Yamashita, Masanobu Ishii, Tatsuya Tokai, So Ikebe, Yoshinori Yamanouchi, Taishi Nakamura, Kenichi Tsujita

**Affiliations:** a Shinbeppu Hospital, Oita, Japan; b Department of Cardiovascular Medicine, Graduate School of Medical Science, Kumamoto University, Kumamoto, Kumamoto, Japan; c Department of Medical Information Science, Graduate School of Medical Science, Kumamoto University, Kumamoto, Japan; d Department of Clinical Investigation, Kumamoto University Hospital, Kumamoto, Japan

**Keywords:** Triglyceride, Cardiovascular disease, Dyslipidemia

## Abstract

**Background:**

Previous analyses have reported that low triglyceride (TG) levels were associated with a reduced risk of cardiovascular events in a primary prevention cohort. However, it remains unclear whether a reduction in TG levels directly contributes to cardiovascular risk reduction.

**Objective:**

To investigate whether a reduction in TG levels is associated with a decreased risk of cardiovascular events in primary and secondary prevention cohorts.

**Methods:**

This retrospective study was conducted with a nationwide health insurance claims database, with medical checkups between January 2005 and August 2020 in Japan. We included patients with baseline TG levels ≥150 mg/dL and classified them into primary or secondary prevention of cardiovascular events. TG levels at one year were used to stratify patients into four groups: low (≤100 mg/dL), normal (100–149 mg/dL), high (150–499 mg/dL), and very high (≥500 mg/dL). The primary outcome was major adverse cardiovascular events (MACE).

**Results:**

In the primary prevention cohort, a reduction TG levels to ≤150 mg/dL was significantly associated with a reduced risk of MACE. No significant association was observed in the secondary prevention cohort. In subgroup analyses stratified by LDL-C target achievement, patients with elevated LDL-C showed a trend toward lower event risk with decreasing TG levels.

**Conclusion:**

A weak association was found between a reduction in TG levels and a reduced risk of cardiovascular events in the primary prevention population. However, prospective, randomized, placebo-controlled, and large cardiovascular outcomes trials are needed to prove that substantial reductions in TG levels correlate with cardiovascular event risk reduction.

## 1 Introduction

The management of dyslipidemia is critically important for the prevention of cardiovascular events. In particular, low density lipoprotein cholesterol (LDL-C) lowering therapy centered on statins has been well established as the mainstay of pharmacological treatment, supported by numerous clinical studies [[1], [2], [3]]. Statins are strongly recommended in international guidelines and have firmly established their position in clinical practice [4,5].

Many global guidelines or scientific statements recommend the use of high dose icosapent ethyl (IPE) [2 × 2g/day] in combination with a statin in high-risk or very high-risk patients with elevated triglyceride levels (fasting triglyceride levels 135-499 mg/dL or 1.52–5.63 mmol/L) to reduce the risk of cardiovascular events [6,7]. Although several observational studies have reported that elevated triglyceride(TG) levels are associated with increased risk of cardiovascular events [[8], [9], [10], [11], [12]], randomized controlled trials (RCT) assessing TG-lowering pharmacotherapies have not provided consisted results [[13], [14], [15]]. While studies such as JELIS [15] and REDUCE-IT [13] demonstrated a reduction in major adverse cardiovascular events (MACE) with icosapent ethyl, it remains unclear whether this effect is solely attributable to TG-lowering therapy.

Previous studies using real-world database have shown a significant association between TG levels and cardiovascular events in primary prevention cohorts [16]. Therefore, it is necessary to investigate whether reducing TG levels can lower the risk of cardiovascular events in primary and secondary prevention cohorts. The purpose of this retrospective analysis was to investigate whether a reduction of TG levels is associated with a lower risk of cardiovascular events in primary and secondary prevention cohorts using a real-world database.

## 2 Patients and methods

### 2.1 Study design, participants, and setting

This study was a retrospective analysis of the Japan Medical Data Center (JMDC) Claims Database, which is a nationwide health insurance claims database. It comprises de-identified individual healthcare records (inpatient, outpatient, dispensing) including demographic details, diagnoses categorized under the International Classification of Databases, 10th Revision (ICD-10) coding, medication information, medical procedures, mortality data, hospitalization events, and annual health checkup data for workplace employees, inclusive of blood pressure and lipid profile laboratory results from various health insurance payers, as well as Specific Health Checkups [[17], [18], [19], [20]]. Between January 2005 and August 2020, 3,824,093 participants who underwent a comprehensive medical checkup with measurement of lipid profiles such as LDL-C, high-density lipoprotein cholesterol (HDL—C), and TG were enrolled, as previously described [16]. Of these, analyzed population (*n* = 3,445,328) was identified after exclusion of participants who had missing values of past histories (*n* = 269,788) and data for antihyperlipidemic medications (*n* = 108,977). Participants were classified into primary (*n* = 3,415,522) and secondary prevention (*n* = 29,806) groups based on their history of cardiovascular diseases such as angina pectoris, acute myocardial infarction and coronary revascularization and cerebrovascular diseases. Among these participants, individuals who developed the outcome prior to the one-year health checkup, those with incomplete follow-up, those who did not undergo the one-year health checkup, and those with baseline TG levels <150 mg/dL at the baseline health checkup were excluded. After these exclusions, 350,959 individuals in the primary prevention group and 4819 in the secondary prevention group had baseline TG levels ≥150 mg/dL and had TG measurements taken at the one-year health checkup (Supplementary Fig. A). Based on TG levels measured during the health checkup at one year, these patients were further classified into three groups: low (<100 mg/dL), normal (100-149 mg/dL), high (150-499 mg/dL), or very high (≥500 mg/dL).

### 2.2 Variables

During the health check-up, blood samples were collected from each participant who had fasted for more than 10 h. Blood laboratory test data, such as TG, LDL-C, HDL—C, glucose, and hemoglobin A1C levels at the initial health checkup, were used for exposure. Following >5 min of seated rest, the brachial blood pressure was measured twice with a > 1-min interval while an average of 2 measurements was used for analysis [17,21]. Hypertension was defined as systolic blood pressure ≥ 140 mmHg, diastolic blood pressure ≥ 90 mmHg, or the use of anti-hypertensive agents [17,21]. Diabetes mellitus was defined as a fasting plasma glucose level of >126 mg/dL or the use of anti-diabetic agents [17,18]. Body mass index (BMI) was calculated as weight (kg) divided by height squared (m^2^). The BMI categories were defined as underweight (BMI <18.5 kg/m^2^), normal weight (BMI 18.5 to <25 kg/m^2^), overweight (BMI 25 to <30 kg/m^2^), and obesity (BMI ≥ 30 kg/m^2^). The original questionnaire regarding smoking and alcohol consumption has been previously reported [22]. Briefly, current smoking status was assessed with the question: “Are you a current regular smoker?” (defined as having smoked 100 or more cigarettes or for at least six months, and currently smoking). Alcohol consumption was assessed by asking. “How often do you drink?” with response options of Rarely, Occasionally, or Everyday.

### 2.3 Outcome

The primary outcome was MACE defined as acute myocardial infarction (AMI) (ICD-10 codes: I21.0, I21.1, I21.2, I21.3, I21.4, and I21.9), a hospitalization for unstable angina (ICD-10 code: I20.0), ischemic stroke (ICD-10 codes: I63.0, I63.1, I63.2, I63.3, I63.4, I63.5, I63.6, I63.8, I63.9, I69.3), and cardiac death [17,23]. Cardiac death included cardiocerebrovascular- or acute heart failure (ICD-10 codes: I50.0 and I50.9)- related death. The secondary outcome was all-cause death. If a participant experienced two or more events, the first was counted as the outcome. These outcomes were assessed from the date of the one-year health checkup to August 2020.

### 2.4 Statistical analyses

Continuous variables were presented as median values (IQR), and categorical variables were presented as frequencies and percentages. Group comparisons were analyzed using the Mann-Whitney *U* test for continuous variables between two groups, the Kruskal-Wallis test for continuous variables among the three groups, as appropriate. Cox proportional hazards regression analysis treating event rates of high group as a reference was used to compute the hazard ratios (HRs) and 95 % confidence intervals (CIs) of clinical outcomes associated with TG levels. Multivariable models were adjusted for potential confounding factors such as age, sex, BMI, current smoking status, hypertension, diabetes mellitus, changes in BMI, HbA1c, LDL-C, and HDL-C from baseline to 1 year, statin use at baseline and at 1 year, and the use of TG-lowering agents. Survival curves were estimated using the Kaplan-Meier method and compared using the log-rank test. Subgroup analyses were conducted according to LDL-C levels defined by management categories and the presence or absence of pharmacological treatment for TG. The LDL-C targets were based on the 2022 Japan Atherosclerosis Society Guidelines for the Prevention of Atherosclerotic Cardiovascular Diseases [24] (Supplemental Fig. B). Patients whose LDL-C levels were above the target were classified as the “high LDL-C group,” and those below the target as the “normal LDL-C group.” Pharmacologic treatment was defined as a prescription of fibrates, icosapent ethyl, and omega-3 fatty acids within 6 months from the index date. In the subgroup analysis concerning pharmacologic treatment, propensity score matching was performed using a 1:1 nearest-neighbor approach with a caliper of 0.2 to balance covariates between groups. At the time of enrollment, 297,449 patients had TG ≥150 mg/dL and were not receiving any TG-lowering medication. Among them, 4479 patients began pharmacologic treatment. Using propensity score matching, 4368 patients were selected from both the treatment and non-treatment groups (Supplemental Fig. C). The level of significance was defined as a two-sided *p*-value of <0.05. All statistical analyses were preformed using R software version 4.0.5 (R Foundation for Statistical Computing, Vienna, Austria. https://www.R-project.org/.) and Python (V. 3.7.11, Python Software Foundation).

## 3 Results

### 3.1 Participants characteristics in primary and secondary prevention

Among the patients with baseline TG levels ≥150 mg/dL in primary prevention group, 350,959 individuals were included, with median age of 47 years, and 91 % were male.

In the secondary prevention with TG levels ≥150 mg/dL, 4819 individuals were included, with median age 56 years, and 95 % were male. Baseline characteristics are summarized in Table 1.

**Table 1:** Baseline characteristics of primary and secondary prevention divided by the triglyceride category groups.

	Primary prevention					Secondary prevention				
	Overall *N* = 350,959	Low (<100 mg/dL) *N* = 38,004	Normal (100-149 mg/dL) *N* = 91,931	High (150-499 mg/dL) *N* = 209,465	Very High (≥500 mg/dL) *N* = 11,502	Overall *N* = 4819	Low (<100 mg/dL) *N* = 370	Normal (100-149 mg/dL) *N* = 1202	High (150-499 mg/dL) *N* = 3064	Very high (≥500 mg/dL) *N* = 183
Age, years	47 (41,54)	46 (40, 53)	48 (41, 55)	48 (41, 54)	47 (41,53)	56 (51,61)	57 (52, 61)	57 (52, 62)	56 (51, 61)	53 (49, 58)
Male, n(%)	318,196 (91.0)	32,704 (86.0)	81,705 (89.0)	192,739 (92.0)	11,017 (96.0)	4601 (95.0)	354 (96.0)	1138 (95.0)	2934 (96.0)	175 (96.0)
BMI, kg/m^2^	25.1 (23.1, 27.6)	24.1 (22.1, 26.5)	24.8 (22.8, 27.3)	25.4 (23.4, 28.0)	25.2 (23.2, 27.8)	26.4 (24.3, 29.1)	26.1 (23.9, 28.4)	26.0 (24.0, 28.8)	26.7 (24.5, 29.2)	27.2 (24.7, 30.3)
BMI category, n(%)										
<18.5	3362 (1.0)	853 (2.2)	949 (1.0)	1390 (0.7)	170 (1.5)	10 (0.2)	1 (0.3)	4 (0.3)	4 (0.1)	1 (0.5)
≥18.5, <25	164,740 (47.0)	21,825 (57.0)	46,324 (50.0)	91,430 (44.0)	5171 (45.0)	1510 (31.0)	135 (36.0)	440 (37.0)	886 (29.0)	49 (27.0)
≥25, <30	140,792 (40.0)	12,475 (33.0)	34,730 (38.0)	88,780 (42.0)	4793 (42.0)	2352 (49.0)	179 (48.0)	533 (44.0)	1556 (51.0)	84 (46.0)
≥30	42,065 (12.0)	2851 (7.5)	9928 (11.0)	27,912 (13.0)	1368 (12.0)	947 (20.0)	55 (15.0)	225 (19.0)	618 (20.0)	49 (27.0)
Waist circumference, cm	88 (83, 94)	85 (80, 91)	87 (82, 93)	89 (84, 95)	88 (83, 94)	92 (86, 99)	91 (86, 97)	91 (86, 98)	92 (87, 99)	99 (87, 100)
SBP, mmHg	126 (116, 135)	124 (114, 134)	125 (116, 135)	126 (117, 136)	128 (119, 138)	127 (118, 137)	127 (118, 137)	125 (117, 136)	127 (118, 138)	130 (120, 140)
DBP, mmHg	80 (72, 87)	78 (70, 86)	79 (72, 87)	80 (73, 88)	82 (75, 89)	80 (73, 88)	80 (72, 86)	80 (72, 87)	81 (73, 88)	83 (75, 88)
Hypertention, n(%)	58,036 (17.0)	4604 (12.0)	14,593 (16.0)	36,764 (18.0)	2065 (18.0)	3681 (76.0)	273 (74.0)	937 (78.0)	2328 (76.0)	143 (78.0)
Diabetes, n(%)	18,579 (5.3)	1526 (4.0)	4575 (5.0)	11,615 (5.5)	863 (7.5)	1369 (28.0)	106 (29.0)	319 (27.0)	859 (28.0)	85 (46.0)
Smoking, n(%)	139,800 (40.0)	13,346 (35.0)	33,666 (37.0)	86,686 (41.0)	6091 (53.0)	1132 (23.0)	70 (19.0)	248 (21.0)	742 (24.0)	72 (39.0)
Alcohol consumption, n(%)										
Daily	116,195 (33.0)	12,819 (34.0)	28,633 (31.0)	68,930 (33.0)	5798 (50.0)	1623 (34.0)	126 (34.0)	371 (31.0)	1036 (34.0)	90 (49.0)
Sometimes	132,903 (38.0)	13,816 (36.0)	35,139 (38.0)	80,420 (38.0)	3517 (31.0)	1576 (33.0)	118 (32.0)	399 (33.0)	1012 (33.0)	47 (26.0)
None	101,861 (29.0)	11,369 (30.0)	28,159 (31.0)	60,135 (29.0)	2187 (19.0)	1620 (34.0)	126 (34.0)	432 (36.0)	1016 (33.0)	46 (25.0)
History of cerebrovascular disease, n (%)	NA	NA	NA	NA	NA	1354 (28.0)	110 (30.0)	324 (27.0)	873 (28.0)	47 (26.0)
History of cardiovascular disease, n (%)	NA	NA	NA	NA	NA	3707 (77.0)	276 (75.0)	937 (78.0)	2347 (77.0)	147 (80.0)
History of hemodialysis, n(%)	484 (0.1)	35 (<0.1)	96 (0.1)	332 (0.2)	21 (0.2)	164 (3.4)	115 (3.5)	39 (3.2)	101 (3.3)	14 (7.7)
Glucose, mg/dL	97 (90, 106)	95 (88, 103)	96 (90, 105)	97 (91, 107)	100 (92, 112)	108 (97, 126)	104 (96, 123)	106 (96, 123)	108 (97, 127)	119 (103, 145)
At baseline										
HbA1c, %	5.50 (5.30, 5.80)	5.50 (5.20, 5.70)	5.50 (5.30, 5.80)	5.59 (5.30, 5.90)	5.50 (5.30, 5.90)	6.00 (5.60, 6.70)	5.90 (5.60, 6.70)	5.90 (5.60, 6.70)	6.00 (5.69, 6.70)	6.30 (5.79, 7.40)
LDL-C, mg/dL	133 (111,155)	127 (105,149)	134 (113, 155)	134 (113, 156)	112 (87,137)	105 (87, 126)	104 (88, 126)	106 (88,125)	105 (87, 126)	103 (76, 124)
HDL-C, mg/dL	48 (42, 56)	53 (45,62)	50 (44, 58)	47 (41, 54)	44 (37,52)	47 (41, 55)	50 (44,59)	49 (42, 57)	46 (41, 54)	43 (38, 51)
TG, mg/dL	198 (169, 255)	176 (160, 208)	179 (162, 211)	213 (177, 273)	390 (266, 584)	201 (170, 263)	172 (157, 213)	180 (162, 214)	214 (178, 276)	420 (302, 589)
Statin therapy, n (%)	917 (1.2)	82 (1.2)	209 (1.0)	590 (1.2)	36 (1.5)	21 (0.5)	4 (1.2)	6 (0.6)	9 (0.3)	2 (1.6)
TG lowering therapy, n (%)	1079 (0.3)	64 (0.2)	179 (0.2)	735 (0.4)	101 (0.9)	41 (0.9)	2 (0.5)	6 (0.5)	31 (1.0)	2 (1.1)
At 1-year follow-up										
BMI, kg/m2	25.1 (23.0, 27.6)	23.5 (21.6, 25.7)	24.6 (22.6, 27.1)	25.5 (23.4, 28.1)	25.5 (23.4, 28.0)	26.4 (24.3, 29.0)	25.3 (23.3, 27.6)	25.8 (23.7, 28.5)	26.7 (24.6, 29.2)	27.5 (24.7, 30.3)
Unknown	28	4	9	15	0	1	0	0	1	0
HbA1c, %	5.50 (5.30, 5.90)	5.40 (5.20, 5.70)	5.50 (5.30, 5.90)	5.60 (5.30, 5.90)	5.60 (5.30, 6.00)	6.00 (5.60, 6.70)	5.80 (5.60, 6.40)	5.90 (5.60, 6.55)	6.10 (5.70, 6.80)	6.30 (5.70, 7.40)
Unknown	27,150	3186	6963	15,996	1005	335	25	90	208	12
LDL-C, mg/dL	133 (111, 155)	125 (105, 147)	135 (115, 156)	134 (113, 157)	96 (75, 119)	104 (86, 124)	99 (81, 117)	103 (87, 122)	106 (87, 126)	95 (67, 120)
Unknown	123	13	19	64	27	3	0	1	2	0
HDL-C, mg/dL	49 (42, 58)	58 (50, 68)	52 (46, 61)	47 (41, 54)	40 (34, 47)	48 (42, 56)	55 (48, 64)	51 (44, 59)	47 (41, 54)	41 (35, 48)
Unknown	30	5	5	15	5	0	0	0	0	0
TG, mg/dL	173 (129, 238)	83 (71, 92)	127 (114, 138)	212(177, 269)	647 (556, 826)	180 (137, 248)	87 (74, 94)	128 (115, 139)	212 (177, 272)	629 (553, 817)
Statin therapy, n (%)	38,451 (11.0)	3918 (10.0)	11,386 (12.0)	23,332 (11.0)	815 (7.1)	3859 (80.0)	312 (84.0)	1006 (84.0)	2441 (80.0)	100 (55.0)
TG lowering therapy, n (%)	18,144 (5.2)	1410 (3.7)	4123 (4.5)	11,405 (5.4)	1206 (10.0)	1189 (25.0)	74 (20.0)	260 (22.0)	774 (25.0)	81 (44.0)

Data are n (%) or median (IQR).

BMI indicates Body mass index; SBP, systolic blood pressure; DBP, diastolic blood pressure; HbA1c, hemoglobin A1c; NA, not available.

### 3.2 Outcome

**Fig. 1:**
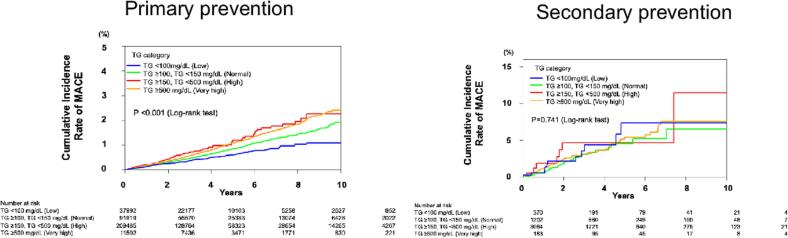
Association between triglyceride levels at one year and cumulative incidence of MACE in primary and secondary prevention Kaplan-Meier survival curves were estimated to evaluate cumulative incidence of MACE and compared using the log-rank test in primary and secondary prevention cohort. MACE, major adverse cardiovascular event; TG, triglyceride.

During a mean follow-up of 3.17 ± 2.56 years, MACE occurred in 2260 patients, AMI in 968 patients, stroke in 756 patients, UAP in 531 patients, cardiac death in 74 in patients, and all-cause death in 720 patients. In primary prevention group, multivariable analysis showed that a reduction in TG levels was independently associated with a reduced risk of MACE and AMI in the low and normal TG group (MACE; HR: 0.73, 95 % CI: 0.60–0.88 for low group. HR: 0.87, 95 % CI: 0.77–0.97 for normal group. AMI; HR: 0.64, 95 % CI: 0.48–0.87 for low group, HR: 0.77, 95 % CI: 0.64–0.91 for normal group) and stroke in the low TG group (HR: 0.67, 95 % CI: 0.48–0.95). However, no significant reduction in UAP, cardiac death or all-cause death was observed (UAP; HR: 1.04, 95 % CI: 0.73–1.49, cardiac death; HR: 0.83, 95 % CI: 0.32–2.16, all-cause death; HR 1.11, CI: 0.84–1.46 for low group. UAP; HR: 1.02, 95 % CI: 0.81–1.29, cardiac death; HR: 0.69, 95 % CI: 0.36–1.33, all-cause death; HR 0.88, CI: 0.72–1.08 for normal group). In very high TG group, no significant increase in events was observed with higher TG levels (MACE; HR: 1.03, 95 % CI: 0.81–1.30, AMI; HR: 1.00, 95 % CI: 0.70–1.43, UAP; HR: 1.08, 95 % CI: 0.66–1.79, cardiac death; HR: 1.37, 95 % CI: 0.41–4.51, all-cause death; HR:0.92, 95 % CI: 0.58–1.47) (Table 2). Kaplan-Meier curves demonstrated a trend toward decreased risk of cardiovascular events in individuals with a reduction in TG levels (Fig. 1).

**Table 2:** Results of multivariable Cox proportional hazard model for outcomes in primary and secondary prevention groups.

	Primary prevention			Secondary prevention		
	Number of patients	Number of events	HR (95 % CI)	Number of patients	Number of events	HR (95 % CI)
MACE						
Low (<100 mg/dL)	37,992	146	0.73 (0.60–0.88)	370	11	1.06 (0.51–2.20)
Normal (100 to 149 mg/dL)	91,919	486	0.87 (0.77–0.97)	1202	29	0.90 (0.57–1.41)
High (150 to 499 mg/dL)	209,485	1406	Ref	3064	86	Ref
Very High (≥500 mg/dL)	11,502	89	1.03 (0.81–1.30)	183	7	1.14 (0.49–2.67)

AMI						
Low (<100 mg/dL)	37,992	61	0.64 (0.48–0.87)	370	4	1.16 (0.33–4.08)
Normal (100 to 149 mg/dL)	91,919	195	0.77 (0.64–0.91)	1202	9	0.98 (0.45–2.14)
High (150 to 499 mg/dL)	209,485	630	Ref	3064	27	Ref
Very High (≥500 mg/dL)	11,502	40	1.00 (0.70–1.43)	183	2	1.20 (0.27–5.27)

UAP						
Low (<100 mg/dL)	37,992	40	1.04 (0.73–1.49)	370	3	0.78 (0.22–2.72)
Normal (100 to 149 mg/dL)	91,919	119	1.02 (0.81–1.29)	1202	14	0.98 (0.50–1.93)
High (150 to 499 mg/dL)	209,485	298	Ref	3064	37	Ref
Very High (≥500 mg/dL)	11,502	18	1.08 (0.66–1.79)	183	4	1.40 (0.41–4.72)

Stroke						
Low (<100 mg/dL)	37,992	47	0.67 (0.48–0.95)	370	4	1.35 (0.35–5.15)
Normal (100 to 149 mg/dL)	91,919	169	0.89 (0.74–1.08)	1202	6	0.55 (0.19–1.64)
High (150 to 499 mg/dL)	209,485	475	Ref	3064	24	Ref
Very High (≥500 mg/dL)	11,502	28	0.93 (0.60–1.43)	183	3	1.51 (0.34–6.81)

Cardiac death						
Low (<100 mg/dL)	37,992	5	0.83 (0.32–2.16)	370	0	NA
Normal (100 to 149 mg/dL)	91,919	15	0.69 (0.36–1.33)	1202	0	NA
High (150 to 499 mg/dL)	209,485	50	Ref	3064	0	Ref
Very High (≥500 mg/dL)	11,502	4	1.37 (0.41–4.51)	183	0	NA

All-cause death						
Low (<100 mg/dL)	37,992	79	1.11 (0.84–1.46)	370	1	2.68 (0.29–24.62)
Normal (100 to 149 mg/dL)	91,919	170	0.88 (0.72–1.08)	1202	8	3.14 (0.88–11.18)
High (150 to 499 mg/dL)	209,485	423	Ref	3064	8	Ref
Very High (≥500 mg/dL)	11,502	29	0.92 (0.58–1.47)	183	2	5.98 (1.06–33.68)

MACE, major adverse cardiovascular events; AMI, acute myocardial infarction; UAP; unstable angina pectoris; HR, hazard ratio; CI, confidence interval.

In the secondary prevention group, contrary to the findings in the primary prevention group, multivariable analyses showed that low and normal groups were not significantly associated with reduce risk of any outcomes, whereas very high group was significantly associated with an increased risk of all-cause death (Table 2). Kaplan-Meier curves did not show a significant reduction in event incidence in this group (Fig. 1).

### 3.3 Subgroup analysis

**Fig. 2:**
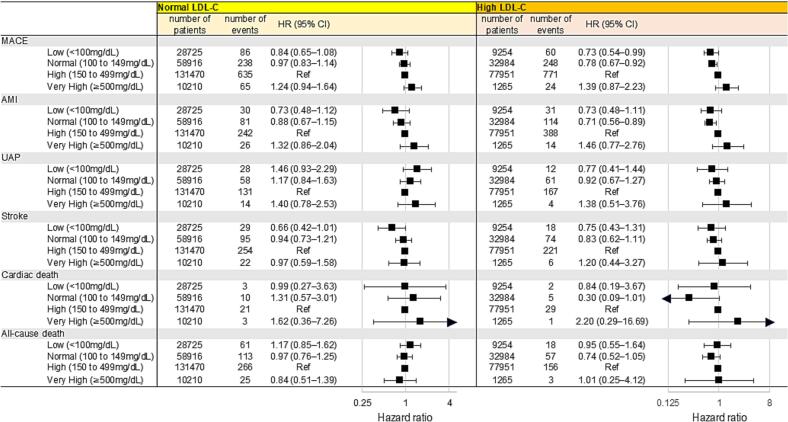
Subgroup analyses according to LDL-C levels defined by management categories The LDL-C targets were based on the 2022 Guidelines for the Prevention of Atherosclerotic Cardiovascular Diseases. Normal LDL-C was defined as LDL-C < 160 mg/dL in low-risk patients, <140 mg/dL in intermediate-risk patients, <120 mg/dL in high-risk patients. In the multivariable model, hazard rations (HRs) were adjusted for age, sex, body mass index, smoking, hypertension, diabetes mellitus, statin use. LDL-C, low density lipoprotein cholesterol; HR, hazard ratio; CI, confidence interval; AMI, acute myocardial infarction. Other abbreviations are listed in Fig. 1.

As shown in Fig. 2, multivariable analysis showed that a decrease in TG levels was not associated with a reduction in cardiovascular events in the normal LDL-C group. In contrast, in the high LDL-C group, a reduction in TG levels were significantly associated with a reduction in MACE (HR: 0.63, 95 % CI: 0.48–0.84 for low TG group; HR: 0.86, 95 % CI: 0.74–0.99 for normal TG group). Change of lipid profile at 1 year in primary prevention was shown in Supplemental Fig. D.

As shown in Supplemental Fig. E, no significant reduction in cardiovascular events was observed with pharmacologic therapy in either the primary or secondary prevention groups. In the secondary prevention cohort, there was a tendency for a higher incidence of cardiovascular events in the pharmacologic treatment group.

## 4 Discussion

Our analysis of a nationwide claims database yielded several key findings: 1) among patients with baseline TG levels ≥150 mg/dL in the primary prevention group, a reduction in TG levels at one year was weakly associated with a decreased risk of MACE on multivariate analysis; 2) in patients with high LDL-C levels, a reduction in TG levels was significantly associated with a reduction in MACE; and 3) pharmacological interventions targeting TG levels were not associated with a reduced risk of MACE in this retrospective analysis of a real-world setting.

Previous observational studies demonstrated that elevated TG levels are associated with increased risk of cardiovascular events [[8], [9], [10], [11], [12]]. However, RCTs have yielded controversial results regarding the efficacy of TG-lowering therapies in high-risk populations for cardiovascular events [[13], [14], [15]]. Therefore, we investigated the association between TG levels and the risk of cardiovascular events using a real-world database [16]. Our findings revealed a significant association between TG levels and cardiovascular event risk in the primary prevention group [16]. Based on these results, we further analyzed whether a reduction in TG levels was associated with a reduced cardiovascular event risk in the present study. This study was designed to stratify patients with elevated TG levels based on their TG levels at one year and to evaluate their cardiovascular event risk. To our knowledge, although there have been meta-analysis demonstrating a reduction in cardiovascular event risk through TG lowering [25], no studies have shown this effect using real-world data with a design similar to ours. This represents the novelty of our study. While no clear benefit of TG reduction was observed in the secondary prevention group, a weak association was found in the primary prevention group. Since cardiovascular events are directly linked to patient prognosis, the observed benefit in primary prevention is particularly meaningful, highlighting the significance of our findings. In the primary prevention group, the present analysis revealed that a reduction in TG levels at one year was associated with the lower risk of MACE. In PROMINENT [13] and STRENGTH [26], where previous RCTs did not show a cardiovascular event risk reduction effect by reduction TG levels, post treatment TG levels were 189 mg/dL, 191 mg/dL, respectively. Both were above 150 mg/dL, and it is conceivable that inadequate TG reduction may not have demonstrated a reduction in cardiovascular event risk. In the AIM-HIGH [27], HPS2-THRIVE [28], and ACCORD-lipid [29] trials, triglyceride levels decreased to below 150 mg/dL in the treatment groups following intervention; however, none of these studies demonstrated a reduction in cardiovascular event risk. These trials, however, included or primarily targeted secondary prevention populations, and patient backgrounds may have influenced the results. Therefore, prospective studies designed with interventions capable of achieving adequate triglyceride lowering and with consideration of patient backgrounds are warranted.

Moreover, pharmacological TG lowering therapy did not show a significant association with reduced MACE risk compared to non-pharmacologic management. In fact, in the secondary prevention group, the incidence of MACE was significantly higher in the pharmacologic treatment group. It is possible that patients at higher risk for MACE were more likely to receive pharmacologic therapy. This could reflect confounding by indication, where high-risk patients were started on medication, rather than a direct effect of the pharmacologic intervention itself. Additionally, information regarding which medications were used for TG control was not specified in the dataset. In previous RCTs, a variety of agents have used for TG-lowering therapy, differences in cardiovascular risk reduction have been observed depending on the drug used. For instance, the PROMINENT trial [13] using pemafibrate did not demonstrate significant benefit, whereas the REDUCE-IT [14], JELIS [15], and RESPECT-EPA [30] trials using icosapent ethyl did show favorable outcomes. It should be noted that the cardiovascular event-reducing effect of icosapent ethyl is thought to involve multiple mechanisms beyond triglyceride reduction, such as antiplatelet activity, suggesting that its benefits may not be solely attributable to lowering triglycerides [31]. These studies targeted patients at high risk for cardiovascular events, such as those with diabetes, dyslipidemia, or a history of cardiovascular disease. As studies of pharmacologic therapy in the primary prevention cohort, VITAL [32] and ASCEND [33] have been conducted in the past, but neither demonstrated a cardiovascular event-reducing effect of omega-3 acid ethyl ester with EPA and DHA. In addition, the ACCORD lipid trial [29], which investigated the effect of fenofibrate in a population in which more than 60 % of patients were in the primary prevention cohort, also failed to show a benefit in reducing cardiovascular events. However, none of these studies evaluated outcomes in the specific subgroup of patients in the primary prevention group whose triglyceride levels decreased from ≥150 mg/dL to <150 mg/dL, in whom our study demonstrated an association with event reduction. Therefore, further research is needed to determine which TG-lowering agents and to what extent TG should be reduced to achieve the greatest clinical benefit.

This study has several limitations. First, its retrospective nature, relying on the JMDC Claims Database, may have introduced a selection bias. The JMDC database primarily consists of data from employer-provided health insurance plans, which over-represent working-age male participants, particularly in the secondary prevention cohort. As a result, our study had a significant gender imbalance and included a preponderance younger patients compared with general high risk of cardiovascular diseases. Moreover, in the secondary prevention group, the number of patients included in the final analysis was extremely small, raising the possibility of substantial selection bias. Secondly, as this was a database study, residual confounding factors may remain. In particular, information on exercise habits and dietary patterns, which have a major impact on triglyceride levels, was not available and could not be adjusted for. Third, although we adjusted for numerous confounding factors, unmeasured confounders may have influenced the observed associations. Fourth, our findings were based mainly on the Japanese population, which may limit their generalizability to other ethnic groups or populations. Fifth, potential underreporting or misclassification of outcomes may have occurred, given the reliance on ICD-10 codes for event identification.

## 5 Conclusion

In our study, among patients in the primary prevention group with TG ≥150 mg/dL, a reduction in TG levels at one year was shown to be weakly associated with a lower risk of cardiovascular events. Further prospective studies are warranted to confirm the degree of TG lowering and to identify specific agents that may reduce cardiovascular risk.

## CRediT authorship contribution statement

**Izuki Yamashita:** Writing – original draft, Validation, Methodology, Investigation, Data curation, Conceptualization. **Masanobu Ishii:** Writing – review & editing, Validation, Formal analysis, Conceptualization. **Tatsuya Tokai:** Visualization, Investigation, Data curation. **So Ikebe:** Investigation, Data curation. **Yoshinori Yamanouchi:** Software, Resources, Data curation. **Taishi Nakamura:** Supervision. **Kenichi Tsujita:** Supervision.

## Ethical statement

This study was conducted in accordance with the principles of the Declaration of Helsinki. As only anonymized clinical data were analyzed, the requirement for informed consent was waived.

## Funding

None.

## Declaration of competing interest

None.

## Appendix A Supplementary data

Supplementary figures Image 1

## References

[1] Baigent C., Blackwell L., Emberson J. (2010). Efficacy and safety of more intensive lowering of LDL cholesterol: a meta-analysis of data from 170,000 participants in 26 randomised trials. Lancet.

[2] Silverman M.G., Ference B.A., Im K. (2016). Association between lowering LDL-C and cardiovascular risk reduction among different therapeutic interventions: a systematic review and meta-analysis. JAMA.

[3] Fulcher J., O’Connell R., Voysey M. (2015). Efficacy and safety of LDL-lowering therapy among men and women: meta-analysis of individual data from 174,000 participants in 27 randomised trials. Lancet.

[4] Vrints C., Andreotti F., Koskinas K.C. (2024). 2024 ESC guidelines for the management of chronic coronary syndromes. Eur. Heart J..

[5] Virani S.S., Newby L.K., Arnold S.V. (2023). 2023 AHA/ACC/ACCP/ASPC/NLA/PCNA guideline for the management of patients with chronic coronary disease: a report of the American Heart Association/American College of Cardiology Joint Committee on clinical practice guidelines. Circulation.

[6] Mach F., Koskinas K.C., Roeters van Lennep J.E. (2025). Focused update of the 2019 ESC/EAS guidelines for the management of dyslipidaemias. Atherosclerosis.

[7] Miller M., Tokgozoglu L., Parhofer K.G. (2022). Icosapent ethyl for reduction of persistent cardiovascular risk: a critical review of major medical society guidelines and statements. Expert. Rev. Cardiovasc. Ther..

[8] Sarwar N., Danesh J., Eiriksdottir G. (2007). Triglycerides and the risk of coronary heart disease: 10,158 incident cases among 262,525 participants in 29 Western prospective studies. Circulation.

[9] Miller M., Stone N.J., Ballantyne C. (2011). Triglycerides and cardiovascular disease: a scientific statement from the American Heart Association. Circulation.

[10] Patel A., Barzi F., Jamrozik K. (2004). Serum triglycerides as a risk factor for cardiovascular diseases in the Asia-Pacific region. Circulation.

[11] Nordestgaard B.G., Langsted A., Mora S. (2016). Fasting is not routinely required for determination of a lipid profile: clinical and laboratory implications including flagging at desirable concentration cut-points-a joint consensus statement from the European Atherosclerosis Society and European Federation of Clinical Chemistry and Laboratory Medicine. Eur. Heart J..

[12] Higashiyama A., Wakabayashi I., Okamura T. (2021). The risk of fasting triglycerides and its related indices for ischemic cardiovascular diseases in Japanese community dwellers: the Suita study. J. Atheroscler. Thromb..

[13] Das Pradhan A., Glynn R.J., Fruchart J.C. (2022). Triglyceride lowering with pemafibrate to reduce cardiovascular risk. N. Engl. J. Med..

[14] Bhatt D.L., Steg P.G., Miller M. (2019). Cardiovascular risk reduction with icosapent ethyl for hypertriglyceridemia. N. Engl. J. Med..

[15] Yokoyama M., Origasa H., Matsuzaki M. (2007). Effects of eicosapentaenoic acid on major coronary events in hypercholesterolaemic patients (JELIS): a randomised open-label, blinded endpoint analysis. Lancet.

[16] Mizuta H., Ishii M., Ikebe S. (2025). Triglycerides and the risk of atherosclerotic cardiovascular events across different risk categories. J. Atheroscler. Thromb..

[17] Kaneko H., Yano Y., Itoh H. (2021). Association of blood pressure classification using the 2017 American College of Cardiology/American Heart Association blood pressure guideline with risk of heart failure and atrial fibrillation. Circulation.

[18] Kaneko H., Itoh H., Kiriyama H. (2021). Lipid profile and subsequent cardiovascular disease among young adults aged < 50 years. Am. J. Cardiol..

[19] Kawasaki R., Konta T., Nishida K. (2018). Lipid-lowering medication is associated with decreased risk of diabetic retinopathy and the need for treatment in patients with type 2 diabetes: a real-world observational analysis of a health claims database. Diabetes Obes. Metab..

[20] Goto A., Goto M., Terauchi Y., Yamaguchi N., Noda M. (2016). Association between severe hypoglycemia and cardiovascular disease risk in Japanese patients with type 2 diabetes. J. Am. Heart Assoc..

[21] Ishii M., Seki T., Sakamoto K. (2020). Association of short term exposure to Asian dust with increased blood pressure. Sci. Rep..

[22] Fukasawa T., Tanemura N., Kimura S., Urushihara H. (2020). Utility of a specific health checkup database containing lifestyle behaviors and lifestyle diseases for employee health insurance in Japan. J. Epidemiol..

[23] Ishii M., Kaikita K., Sakamoto K. (2020). Characteristics and in-hospital mortality of patients with myocardial infarction in the absence of obstructive coronary artery disease in super-aging society. Int. J. Cardiol..

[24] Okamura T., Tsukamoto K., Arai H. (2024). Japan atherosclerosis society (JAS) guidelines for prevention of atherosclerotic cardiovascular diseases 2022. J. Atheroscler. Thromb..

[25] Marston N.A., Giugliano R.P., Im K. (2019). Association between triglyceride lowering and reduction of cardiovascular risk across multiple lipid-lowering therapeutic classes: a systematic review and meta-regression analysis of randomized controlled trials. Circulation.

[26] Nicholls S.J., Lincoff A.M., Garcia M. (2020). Effect of high-dose Omega-3 fatty acids vs corn oil on major adverse cardiovascular events in patients at high cardiovascular risk: the STRENGTH randomized clinical trial. Jama.

[27] Boden W.E., Probstfield J.L., Anderson T. (2011). Niacin in patients with low HDL cholesterol levels receiving intensive statin therapy. N. Engl. J. Med..

[28] Landray M.J., Haynes R., Hopewell J.C. (2014). Effects of extended-release niacin with laropiprant in high-risk patients. N. Engl. J. Med..

[29] Ginsberg H.N., Elam M.B., Lovato L.C. (2010). Effects of combination lipid therapy in type 2 diabetes mellitus. N. Engl. J. Med..

[30] Miyauchi K., Iwata H., Nishizaki Y. (2024). Randomized trial for evaluation in secondary prevention efficacy of combination therapy-statin and Eicosapentaenoic acid (RESPECT-EPA). Circulation.

[31] Mourikis P., Benkhoff M., Wildeis L. (2025). Icosapent ethyl reduces arterial thrombosis by inhibition of cyclooxygenase-1-induced platelet reactivity. Sci. Transl. Med..

[32] Manson J.E., Cook N.R., Lee I.M. (2019). Marine n-3 fatty acids and prevention of cardiovascular disease and cancer. N. Engl. J. Med..

[33] Bowman L., Mafham M., Wallendszus K. (2018). Effects of n-3 fatty acid supplements in diabetes mellitus. N. Engl. J. Med..

