# Supplementary material for: Association between a reduction in triglyceride levels and risk of cardiovascular events
Source: Am Heart J Plus. 2025 Oct 16;60:100647. doi: 10.1016/j.ahjo.2025.100647 (PMC12569805; doi:10.1016/j.ahjo.2025.100647)
Supplement: Supplementary file 1 — Supplementary figures [file mmc1.docx]

**Supplemental Online Content**

**Yamashita I, Ishii M, et al. Association between a reduction in triglyceride levels and risk of cardiovascular events**

**Figure A.** Flow Diagram for Inclusion

**Figure B.** The LDL-C targets based on the 2022 Japan Atherosclerosis Society Guidelines for the Prevention of Atherosclerotic Cardiovascular Diseases

**Figure C.** Flow Diagram for Inclusion of Subgroup Analysis Concerning Pharmacologic Treatment

**Figure D.** Change of Lipid Profile at One Year in Primary Prevention

**Figure E.** Subgroup Analyses According to the Presence or Absence of Pharmacological Treatment for Triglycerides.

**Figure A. Flow Diagram for Inclusion**

MACE, major adverse cardiovascular event; TG, triglyceride

.

**Figure B. The LDL-C targets based on the 2022 Japan Atherosclerosis Society Guidelines for the Prevention of Atherosclerotic Cardiovascular Diseases**

Target levels for low-density lipoprotein cholesterol (LDL-C) according to current clinical practice guidelines. This figure summarizes the recommended target levels for LDL-C based on the latest versions of the Japan Atherosclerosis Society. Values are stratified by cardiovascular risk categories as defined by the guideline.

DM, diabetes mellitus; CKD, chronic kidney disease; PAD, peripheral artery disease; LDL-C, low density lipoprotein cholesterol; HDL-C, high density lipoprotein cholesterol; ASCVD, atherosclerotic cardiovascular disease.

**Figure C. Flow Diagram for Inclusion of Subgroup Analysis Concerning Pharmacologic Treatment**

Abbreviations are listed in Figure A and B.

**Figure D. Change of Lipid Profile at One Year in Primary Prevention**

. Other abbreviations are listed in Figure A.

**Figure E. Subgroup Analyses According to the Presence or Absence of Pharmacological Treatment for Triglycerides.**

CI, confidence interval; PS, propensity score; IPTW, inverse probability of treatment weighting; aHR, adjusted hazard ratio
